# Maternal nutrient restriction in the ewe from early to midgestation programs reduced steroidogenic enzyme expression and tended to reduce progesterone content of corpora lutea, as well as circulating progesterone in nonpregnant aged female offspring

**DOI:** 10.1186/1477-7827-11-34

**Published:** 2013-05-08

**Authors:** Nathan M Long, Nuermaimaiti Tuersunjiang, Lindsey A George, Caleb O Lemley, Yan Ma, William J Murdoch, Peter W Nathanielsz, Stephen P Ford

**Affiliations:** 1 The Center for the Study of Fetal Programming, Laramie, WY 82071, USA; 2 Department of Animal and Veterinary Sciences, Clemson University, Clemson, SC 29634, USA; 3 Department of Animal Science, University of Wyoming, Laramie, WY 82071, USA; 4 Department of Animal and Dairy Sciences, Mississippi State University, Starkville, MS 39762, USA; 5 Department of Obstetrics and Gynecology, University of Texas Health Sciences Center, San Antonio, TX 78229, USA

**Keywords:** Fetal programming, Maternal nutrient restriction, Altered progesterone levels, Aged female offspring, Sheep

## Abstract

**Background:**

Previously we reported decreased circulating progesterone and fertility in one and two year old ewes born to undernourished mothers. This study was designed to investigate if this reduction in progesterone persisted into old age, and if it did, what mechanisms are involved.

**Methods:**

Ewes were fed a nutrient restricted (NR, 50% of NRC recommendations) or control (C, 100% of NRC) diets from day 28 to 78 of gestation, then all were fed to requirements through parturition and weaning. Female offspring (4 per treatment group) were maintained as a group and fed to requirements from weaning until assigned to this study at 6 years of age. Ewes were synchronized for estrus (day 0) and blood samples were collected daily from day 0 to day 11 before necropsy on day 12. Blood serum and luteal tissue were assayed for progesterone concentrations by validated radioimmunoassay.

**Results:**

Circulation progesterone concentrations tended to be lower (*P* = 0.06) in NR than C offspring from day 0 to 11 of the estrous cycle. While total luteal weight was similar across groups, total progesterone content also tended to be reduced (*P* = 0.07) in luteal tissue of NR than C offspring. Activity of hepatic progesterone catabolizing enzymes and selected angiogenic factors in luteal tissue were similar between groups. Messenger RNA expression of steroidogenic enzymes StAR and P450scc were reduced (*P* < 0.05), while protein expression of StAR tended to be reduced (*P* < 0.07) and P450scc was reduced (*P* < 0.05) in luteal tissue of NR versus C offspring.

**Conclusions:**

There appears to be no difference in hepatic steroid catabolism that could have led to the decreased serum progesterone. However, these data are consistent with the programming of decreased steroidogenic enzyme expression in CL of NR offspring, leading to reduced synthesis and secretion of progesterone.

## Background

Early pregnancy loss in livestock is a serious economic problem and has been attributed to insufficient circulating progesterone concentrations [1,2]. It has been reported that female offspring born to ewes undernourished during late gestation exhibit decreased circulating progesterone concentrations [3]. Further, maternal nutrient restriction in ewes during early and mid-gestation resulted in decreased ovulation rate in their female offspring [4]. This defect in ovulation may have been initiated in utero, as we have previously demonstrated that a global 50% nutrient restriction from early to mid-gestation in the ewe increases oxidative base lesions within DNA of oogonia recovered from ovaries of mid-gestation fetuses when compared to those of midgestation fetuses of ewes fed to requirements [5]. We have shown that female offspring born to these nutrient restricted (NR) ewes exhibited reduced circulating progesterone throughout their estrous cycles at both one and two years of age compared to offspring of control (C) ewes [6]. In addition, a marked decrease in lambing rates was observed in these nutrient restricted ewes compared to ewes fed to requirements [6]. However, whether reduction in circulating progesterone concentration persists in the female offspring of nutrient restricted ewes throughout life has not been determined.

The level of circulating progesterone is affected by its clearance induced by progesterone catabolic enzymes in the liver, and ovarian progesterone production by steroidogenic enzymes within the cells of the corpus luteum (CL). In the liver, the activity of cytochrome P450 2C (CYP2C), cytochrome P450 3A (CYP3A), aldo-keto reductase 1C (AKR1C) and cytochrome P450 reductase (P450) are largely responsible for progesterone clearance [7,8]. It is well known that steroidogenic acute regulatory enzyme (StAR), P450 side chain cleavage enzyme (P450scc) and 3β-hydroxyl steroid dehydrogenase (3β-HSD) are the three enzymes most important in controlling the rate of progesterone production, and alterations in expression of these enzymes have been implicated in maternal programming of offspring disorders [9-11]. Other factors impacting progesterone production by the CL are angiogenic factors such as vascular endothelial growth factor (VEGF), fibroblast growth factor (FGF), and angiopoieten 1 and 2 (ANG-1 and ANG-2) which play important roles in stimulating CL vascularization [12-14]. Further, reactive oxygen species such as nitric oxide and hydrogen peroxide have been known to inhibit steroidogenesis by blocking cholesterol transport to mitochondria, and leading to reduced P4 production in the ovary [15,16]. Peroxiredoxin-3 (PRX-3) is a mitochondrial member of the antioxidant thioredoxin peroxidases that reduce mitochondrial hydrogen peroxide [17], and has been utilized as an indicator of mitochondrial oxidative stress. PRX-3 expression is induced by oxidants and is thought to play a role in the antioxidant defense system and homeostasis within mitochondria.

In the current study, we confirmed that progesterone concentrations continued to be reduced during the estrous cycle of aged female offspring (6 years old) born to nutrient restricted versus control fed dams previously reported to have reduced progesterone concentrations as yearlings and two-year olds. Further, we evaluated whether ovarian secretion or hepatic catabolism contribute to the observed group differences in circulating progesterone concentrations.

## Methods

### Feed treatments and animal management

All experiments and procedures were approved by the University of Wyoming Animal Care and Use Committee. The present study was conducted at the Center for the Study of Fetal Programming at the University of Wyoming in Laramie, WY. Following breeding in the fall of 2002, dams were moved to an indoor confinement facility with individual neighboring pens (2 × 3.5 m) with free access to water. Dams were assigned in equal groups to either control (C; 100% of National Research Council [NRC] recommendations; n=4) or nutrient restriction (NR; 50% of NRC recommendations; n=4) diets beginning on day 28 of gestation [18]. Both groups’ diets were made up of a beet pulp-based pellet and all ewes were fed individually each morning. At day 78 of gestation (mid-gestation), NR dams were returned to 100% of recommendations for the remainder of gestation but all dams remained indoors in confinement pens through lambing in spring of 2003. Upon lambing, ewes and lambs from both nutritional groups were moved to an outdoor group pen and fed a diet of hay supplemented with cracked corn. Ewe body weight (BW) and body condition score (BCS) during the experiment has been previously published for this group of female offspring [6]. All lambs were reared by their own dams and weaned at approximately four months of age. Lambs also had free access to a commercially available creep feed (Lamb Creep B30 with Bovatec; Ranch-Way Feeds, Ft. Collins, CO) from 2 weeks of age. After weaning, all female C and NR offspring (singleton) were comingled in a single group and fed to NRC requirements to maintain appropriate body weight and condition through the time of testing at six years of age (2009). These C and NR first filial generation (F1) female offspring were also bred in the intervening years to produce lambs in 2005, 2006, and 2007 as 3, 4 and 5 year olds to a single intact ram, respectively. Ewes were not bred in 2008 for lambing in 2009, the breeding year immediately preceding the initiation of the current study.

### Blood sampling of offspring

In the summer of 2009, the remaining 6 year old female offspring from NR (n=4) and C (n=4) dams were fed an ad libitum diet of a highly palatable pelleted feed for a period of approximately four months. In the final few weeks of feeding, NR and C ewe offspring were injected twice, nine days apart with prostaglandin-F2α (Lutalyse, Pharmacia and Upjohn Co., Kalamazoo, MI) to synchronize estrus. Estrus was then detected by observing ewes for approximately 30 minutes, twice daily with a vasectomized ram. The day copulation was observed was recorded as day 0 of the estrous cycle and daily blood sampling was initiated. Blood sampling for all ewes was initiated within 48 hours of the second prostaglandin injection when visual estrus was detected. Ewes were then bled daily at 0700 hours until the mid-luteal phase of their cycle (day 11). Blood was collected by jugular venipuncture into evacuated blood collection tubes (BD Vacutainer, Franklin Lakes, NJ). Blood was allowed to coagulate approximately one hour at room temperature and then refrigerated at 4°C overnight before centrifugation at 2500 × g for 20 minutes. Serum was aliquoted and stored at -20°C pending hormone analysis.

### Tissue collection

Following daily blood collections through day 11 of the estrous cycle, each ewe was necropsied on day 12 for collection of body tissues. Ewes were sedated with Ketamine (10 mg/kg body weight) and maintained under isoflourane inhalation anesthesia (4% induction, 1-2% maintenance). Ewes were then exsanguinated via the jugular and carotid blood vessels while under general anesthesia and the liver and ovaries quickly removed and weighed. Hepatic tissue was then collected from the left lobe, immediately snap frozen in liquid nitrogen and stored at -80°C pending analysis. Each corpus luteum (CL) was removed from the ovaries and weighed, and then cut into 2 equal portions. One portion was reweighed and placed in a vial containing methanol for subsequent extraction of progesterone. The remaining portion of each CL was snap frozen at -80°C for subsequent mRNA and protein quantitation.

### Progesterone content of luteal tissue and serum concentrations

Progesterone concentrations of both luteal tissue (wet weight-basis) according to validated methods [19] and serum were determined using a commercially available radioimmunoassay kit (Siemens Healthcare Diagnostics, Deerfield, IL) previously validated for use in sheep [6]. Intra-assay coefficients of variation for progesterone assays in luteal tissue and serum were 3.9 and 4.1%, respectively using previously known serum samples that were high and low in progesterone concentrations with duplicates ran at the beginning and end of each assay.

### Hepatic cellular fractionation and enzymatic activity

Frozen liver samples were submerged in 0.1 M phosphate buffer and homogenized using a dounce homogenizer (Kontes-Kimble Chase, LLC). Homogenized tissue was centrifuged at 10,000 × g for 10 minutes and supernatant was collected for experiments using cytosolic fractions. Microsomes were collected and concentrated using differential centrifugation techniques [20]. Homogenized tissue was centrifuged at 10,000 × g for 10 minutes. Pellets were discarded and the supernatants were centrifuged at 100,000 × g for 60 minutes. The microsomal pellets were resuspended in phosphate buffer containing 20% glycerol. Cytosolic and microsomal protein was determined using a Coomassie Plus (Bradford) protein assay following the manufacturer’s protocol (Thermo Scientific, Rockford, IL) and used to standardize cytochrome P450 2C (CYP2C), cytochrome P450 3A (CYP3A), aldo-keto reductase 1C (AKR1C) and cytochrome P450 reductase (P450). Activity of AKR1C was determined in cytosolic cellular fractions using the specific substrate, 1-acenapthenol (Sigma Chemical Co.), following the methods of Savlik et al. [21] and Palackal et al. [22]. Briefly, AKR1C enzymatic reactions contained 150 to 650 μg of cytosolic protein, 250 μM 1-acenapthenol and 500 μM NADP. We used a range of protein concentrations from liver homogenates to test the linearity of AKR1C activity during a sample dilution series (decreasing protein concentration). It was determined that AKR1C activities (the rates of 1-acenapthenol dependent reduction of NADP) was linear with protein concentrations from 100 to 1,000 μg; therefore, we analyzed the AKR1C activities within this linear range and then report activity standardized to the exact amount of protein added to each enzymatic reaction (i.e. pmol of substrate per minute per mg of cytosolic protein). For example as we add lower amounts of protein from the same sample we do not see a decrease in AKR1C activity (activity remains similar) because this activity is standardized relative to the exact amount of protein added to each well. The 1-acenapthenol-dependent reduction of NADP was standardized using the amount of cytosolic protein. Activity of CYP2C and CYP3A was assessed on frozen liver samples following our previously published protocol [23]. Activity of CYP2C was measured as the non-ketoconazole-inhibitable, omeprazole-dependent oxidation of NADPH. Microsomes were pre-incubated for 15 min with 250 μM ketoconazole. Enzymatic reactions for CYP2C contained CYP3A inhibited microsomes, 2.5 mM omeprazole and 250 μM NADPH. Activity of CYP3A was measured as the nifedipine-dependent oxidation of NADPH. Enzymatic reactions for CYP3A contained microsomes, 200 μM nifedipine and 250 μM NADPH. All solutions were added to UV star 96-well plates (PGC Scientifics, Frederick, MD) and the oxidation of NADPH or reduction of NADP was determined by measuring the amount of light absorbed at 340 nm at 37°C for 15 minutes. All reactions were determined to be linear for the entire 15 minutes utilized for calculating activity. The extinction coefficient for NADPH (6220 l/mol*cm) was used to calculate the rate of oxidized NADPH or reduced NADP in pmol/minute*mg of protein. Enzyme activity of P450 was determined in microsomal fractions following the manufactures protocol (product number CY0100; Sigma Chemical Co.) and standardized using microsomal protein concentrations.

### Quantitative RT-PCR

Total RNA was extracted from individual pulverized CL samples using Trizol reagent (Invitrogen Corp., Carlsbad, CA) and then purified by RNeasy mini column (QIAGEN Inc., Valencia, CA) as previously described [24,25]. All RNA samples (n = 4) used in this study were treated with DNase prior to cDNA synthesis. Equal amounts of purified RNA from each CL sample within an animal were pooled for analysis to represent the average expression of mRNA for all CLs in each animal as the number of CL in the ewes was different. Prior to pooling RNA samples from each CL, we confirmed that different sized CLs from the same animal had similar expression of mRNA for the genes examined in this study. Two micrograms of purified RNA sample was used to synthesize single-strand DNA using Promega ImProm- II Reverse Transcription System (Promega Bio-Sciences, San Luis Obispo, CA), according to the kit protocol. Primer sequences for StAR, 3β-HSD, P450scc, peroxiredoxin 3 (PRX-3) and 18s RNA were previously published [25-28]. All Real-time PCR reactions were conducted using a Bio-Rad IQ5 Realtime-PCR Reaction System (Bio-Rad Laboratories Inc., Hercules, CA) as previously utilized in our laboratory [25]. Reactions for each gene were run in duplicate. A temperature gradient PCR reaction was run for all of the primer sets to determine the optimal annealing temperatures. According to gradient PCR, the optimal annealing temperature of all primer sets overlapped at 60°C. Correspondingly, the following protocol was designed and applied to all real-time PCR reactions: I) 1 cycle at 95°C for 3 minutes; II) 40 repeat cycles at 95°C for 15 seconds, followed by annealing at 60°C for 60 seconds; III) 60.0°C-95.0°C with melting temperature increasing 0.5°C for each 30 seconds. Fluorescence was detected at both step II and III. Real time analysis was enabled at step II, and melt curve data collection and analysis enabled at step III. An amplification efficiency of 90-105% is considered to be high in an optimized RT-PCR reaction, and the efficiency of 95 – 100% was reached for all the RT-PCR reactions in this study. Final data was analyzed through the 2-ΔΔCt method where 18 s rRNA was used as a reference gene to normalize all the selected gene expression data.

### Western blotting

Anti-vascular endothelial growth factor (VEGF, rabbit IgG), anti-angiopoietin-1 (ANG-1, goat IgG) and angiopoietin-2 (ANG-2, goat IgG), anti-StAR (goat IgG), and secondary anti-goat IgG antibodies were purchased from Santa Cruz Biotechnology (Santa Cruz, CA). Anti- fibroblast growth factor 2 (FGF-2) antibody (rabbit IgG) was purchased from R&D Systems (Minneapolis, MN). Anti-PRX-3 antibody (rabbit IgG) was purchased from Abcam Cambridge, MA). Secondary anti-rabbit IgG and anti- β-actin (rabbit IgG) antibodies were purchased from Cell Signaling Technology (Danvers, MA). Anti-P450scc (rabbit IgG) and anti-3β-HSD (goat IgG) antibodies were kindly provided by Dr. Walter Miller (University of California, San Francisco, CA) and Dr. J Ian Mason (University of Edinburgh, UK), respectively.

Western blotting was accomplished by methods previously described and utilized in our laboratory [24,25]. Individual CL samples were pulverized in liquid nitrogen and then 0.1 g of each sample was homogenized in a polytron homogenizer with 400 μl of ice-cold buffer containing 137 mM NaCl, 50 mM Tris–HCl, 2% SDS, 1% Triton -100 solution, 10% glycerol, 2.5 mM EDTA, 1 mM CaCl2, 1 mM MgCl2, 2 mM Na3VO4, 100 mM NaF, and 1% protease inhibitor cocktail (Sigma, St. Louis, MO), pH 7.4. Each homogenate was mixed with 2 × standard SDS sample loading buffer and boiled at 95°C for 5 minutes. Equal amounts of protein extract from all CL samples from an individual animal were pooled for western analysis. The final concentration of protein was 10 μg/μl, and 5 μl of lysate was used for loading. A standard SDS-PAGE was run to separate proteins followed by the transfer of separated proteins to a nitrocellulose membrane. Skim milk (5% (wt/vol) dissolved in 0.05% TBST) was used for blocking the membranes, and then the membranes were incubated in primary antibodies diluted in 5% (wt/vol) skim milk over night at 4°C. Primary antibodies for VEGF, FGF-2, ANG-1, and ANG-2 were used at dilutions of 1:200, 1:400, 1:400, and 1:400, respectively. Primary antibodies for StAR, P450scc, 3β-HSD, PRX-3 and β-actin were used at dilutions of 1:500, 1:5000, 1:3000, 1:2000, and 1:1000, respectively. All secondary antibodies were used at a dilution of 1:2,000 in 2% (wt/vol) skim milk. The membrane was then visualized using ECL Western blotting detection reagents and exposed to film. The density of bands was quantified by using an Imager Scanner II (Amersham Biosciences, Piscataway, NJ) and ImageQuant TL software (Amersham Biosciences). The target gene band density was normalized according to the density of β-actin content in the same samples.

### Statistical analysis

The relatively low number of ewes per treatment group (n=4) available for use in this study at 6 years of age, is a limitation, and should be taken into account when assessing the data presented. Repeated variables were analyzed using PROC MIXED in SAS (SAS Institute Inc., Cary, NC) with repeated measures and ewe within treatment as the error term. The model contained time, treatment and their interaction. Differences in non-repeated variables were determined using analysis of variance by PROC GLM in SAS with treatment in the model statement. Mean separation was determined using LSMEANS with PDIFF in SAS following a significant (P ≤ 0.05) preliminary F-test. Differences were considered significant at P ≤ 0.05, tendencies at P ≤ 0.10.

## Results

**Figure 1:**
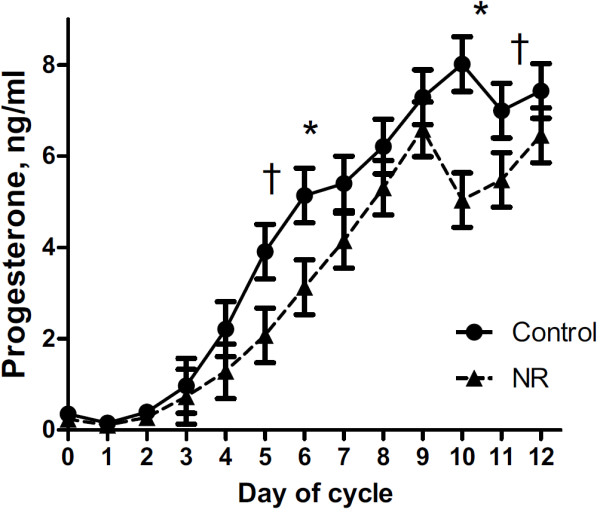
**Daily progesterone concentrations into the mid**-**luteal phase of the estrous cycle in estrus synchronized aged offspring of nutrient restricted and control ewes.** Control (n=4; -●-) ewes and nutrient restricted (n=4; -▲-) offspring. Differences are noted; *, *P* < 0.05 and †, *P* < 0.10.

**Figure 2:**
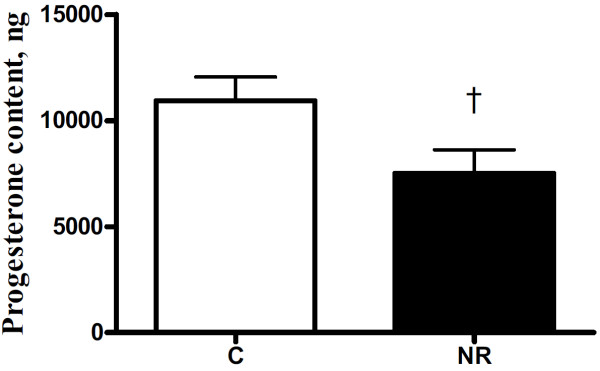
**Total progesterone content of luteal tissue in aged ewe offspring of control and nutrient restricted dams.** Control (n=4, open bars) and nutrient restricted (n=4, solid bars) offspring. Differences are noted; †, *P* < 0.10.

**Figure 3:**
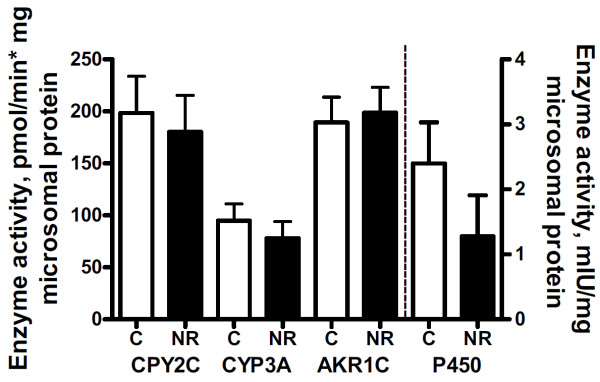
**Enzyme activities from livers of aged ewe offspring of control and nutrient restricted dams for hepatic enzymes involved in progesterone metabolism: cytochrome P450 2C (CYP2C), cytochrome P450 3A (CYP3A), aldo-keto reductase 1C (AKR1C) and cytochrome P450 reductase (P450).** Control (n=4, open bars) and nutrient restricted (n=4, solid bars) offspring. No treatment differences were detected. The dashed line separates activities to be read on the left versus right y-axes. There were no significant effects of treatment on the activity of any enzyme.

Serum progesterone concentrations increased over time from estrus to the mid-luteal phase of the estrous cycle (*P* < 0.01) in all ewes and tended to be lower overall in NR versus C F1 ewe offspring (*P* = 0.06; Figure 1). Serum Progesterone concentration were significantly greater (*P* < 0.05) on day 6 and 10 during the estrous cycle. Total weight and numbers of CL removed from the ovary was not different between NR and C offspring (P > 0.10; Table 1). However, progesterone content of luteal tissue tended to be different between treatment group with NR offspring having lower (*P* = 0.07) total progesterone content than C offspring (Figure 2). There were no group differences (*P* > 0.10) in the activity of any of the four hepatic enzymes involved in progesterone clearance (Figure 3).

**Table 1:** **Ovarian weight**, **corpora lutea** (**CL**) **weight and CL number of aged ewe offspring of control and nutrient restricted dams**

	**Control**	**Nutrient restricted**	**P**-**value**
*n*	4	4	
*Ovarian weight*, *g*	5.48 ± 0.35	5.05 ± 0.46	0.48
*Total CL weight*, *g*	1.30 ± 0.13	1.29 ± 0.16	0.99
*Total CL number*	2.3 ± 0.3	2.3 ± 0.3	0.99

Data are means ± standard error.

Lambing rates were 100% for both NR and C ewe offspring in 2007 (Age 4) and 2008 (Age 5). Also, number of lambs produced per ewe (single vs. twin) was not different (*P* > 0.10) between treatment in either lambing year (Age 4: C, 1.5 ± 0.3 vs. NR, 1.3 ± 0.03 lambs per ewe; Age 5: C, 1.8 ± 0.3 vs. NR, 1.8 ± 0.3 lambs per ewe). However, the total number of lambs produced during the 3 years of lambing was greater (*P* < 0.05) in C ewe offspring compared to NR ewe offspring (4.8 ± 0.6 vs. 3.5 ± 0.6 lambs per ewe) due to the failure of these 4 NR ewes to lamb during their first lambing season as 3 year olds [6].

**Figure 4:**
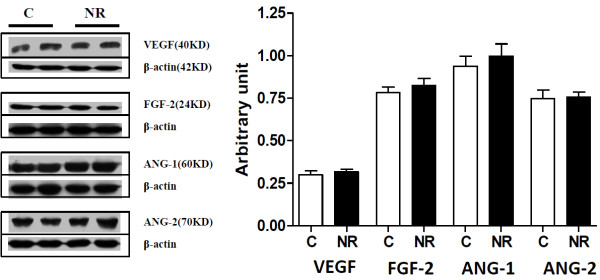
**Protein expression of vascular endothelial growth factor (VEGF), basic fibroblast growth factor (FGF-2), angiopoietin-1 (ANG-1) and angiopoietin-2 (ANG-2) in corpora luteal tissue from aged female offspring of control and nutrient**-**restricted dams.** Control (n=4, open bars) and Nutrient-restricted (n=4, solid bars) offspring.

**Figure 5:**
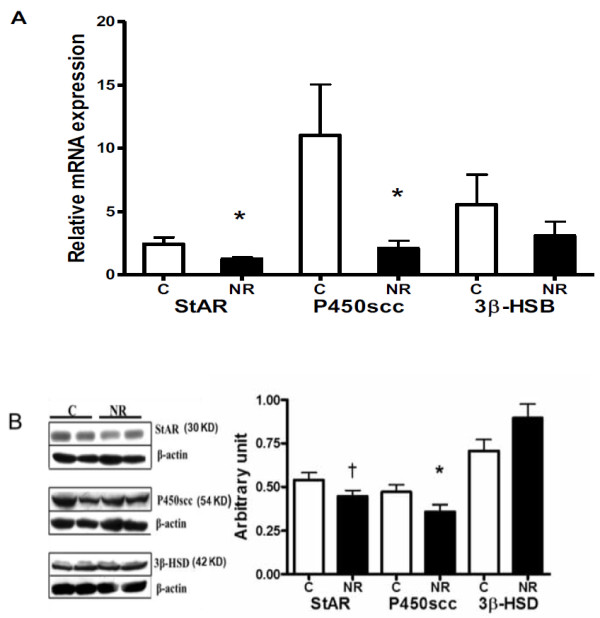
**Message RNA (A) and protein Levels (B) of steroidogenic acute regulator (StAR) enzyme, P450 side chain cleavage (P450scc) enzyme and 3-β Hydroxysteriod dehydrogenase (3β-HSD) in corpora luteal from aged female offspring of control and nutrient-restricted dams.** Control (n=4, open bars) and nutrient-restricted (n=4, solid bars) offspring. Differences are noted; *P < 0.05; †*P* < 0.10.

**Figure 6:**
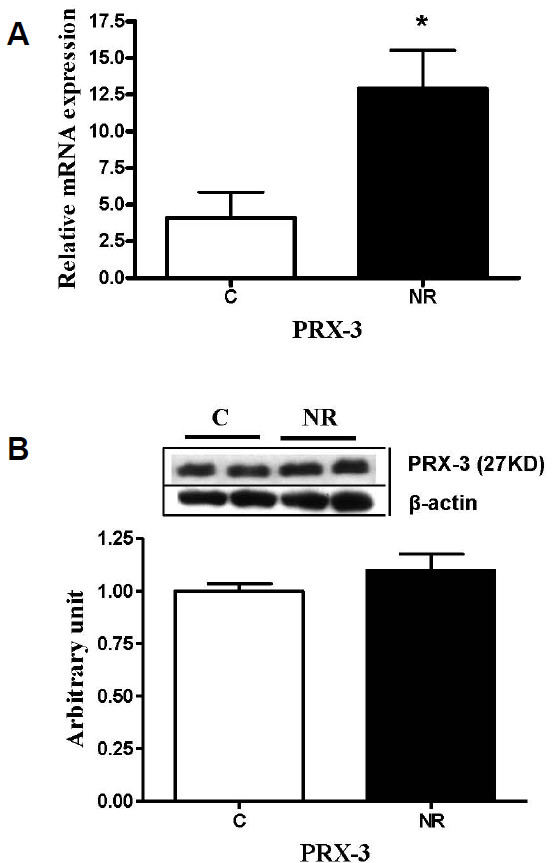
**Message RNA (A) and protein Levels (B) of peroxiredoxin 3 (PRX-3) in corpora luteal from aged female offspring of control and nutrient-restricted dams.** Control (n=4, open bars) and nutrient-restricted (n=4, solid bars) offspring. Differences are noted; *P < 0.05.

Protein expression of luteal angiogenic factors including VEGF, FGF-2, ANG-1 and ANG-2 were similar (*P* > 0.1) between C and NR offspring (Figure 4). The mRNA expression of StAR and P450scc were decreased (*P* < 0.05) in luteal tissue from NR versus C offspring; while 3β-HSD mRNA remained similar (*P* > 0.1) between groups (Figure 5A). Protein expression of StAR tended to be lower (*P* < 0.07), and P450scc protein expression was decreased (P < 0.05) in NR compared to C offspring; protein expression of 3β-HSD was similar across groups (Figure 5B). The mRNA expression of PRX-3 was higher (*P* < 0.05) in NR offspring than in C offspring (Figure 6A); but the protein expression of PRX-3 remained similar (*P* > 0.1) between the two groups (Figure 6B).

## Discussion

These data suggest a programmed effect of decreased circulating progesterone concentrations during the estrous cycle in six year old ewes born to dams experiencing nutrient restriction in the first half of gestation. This effect of decreased circulating progesterone persisted in these NR offspring from one year of age [6] through 6 years of age as observed in the current study, suggesting an apparent permanent programming effect. Furthermore, these data indicate that differences in progesterone concentrations are likely due to differences in ovarian progesterone production rather than clearance of circulating progesterone by specific hepatic enzymes involved in catabolism of progesterone or differences in the number of ovulations, or CL vascularization.

Impaired fertility of farm animals contributes to a lack of productivity in livestock operations, often leading to the culling of such animals from a producers’ flock. In addition, the reduction in lifetime lamb production per ewe by NR offspring compared to the C offspring is also an economic loss for producers. Progesterone plays a major role in the successful establishment and maintenance of pregnancy in domestic ruminant species. Adequate concentrations of circulating progesterone are vital to the successful establishment of pregnancy in sheep and cattle [29-31]. In previously published research, these NR offspring were reported to have reduced progesterone concentrations at one and two years of age [6]. In their first breeding year as 2 year olds (lambing as 3 year olds), these NR offspring also exhibited lower lambing rates than C ewes (14% in NR vs. 100% in C), indicating that decreased progesterone was associated with lower overall fertility of nulliparous female offspring exposed to maternal nutrient restriction during this first pregnancy [6]. We have previously reported an increase in oxidative base lesions within DNA of mid-gestational (Day 78) fetal oogonia in fetuses of NR ewes compared with those of control ewes [5]. Thus, the reproductive failure in NR female offspring in the first pregnancy could also be due to underdeveloped ovarian function in these animals at their first breeding cycle. Meikle at al. [32] reported a decrease in reproductive success of female mice offspring born to undernourished dams throughout gestation. The tendency for persistent reduction of systemic progesterone concentrations throughout the estrous cycle in these NR offspring into older age in this study suggests a permanent programming effect. In a similar sheep model [4], the authors found a decrease in ovulation rate in female offspring born to dams nutrient restricted from early to mid-gestation. An impact of maternal undernutrition on male offspring has also been reported, in that male lambs exposed to maternal undernutrition during mid and late gestation exhibited impaired testicular development [33].

Circulating progesterone levels are regulated by both steroid production and catabolism. Therefore, we examined luteal tissue from the mid-luteal phase at necropsy as it is the primary source for cyclic progesterone production, as well as hepatic enzyme activity which would affect rate of catabolism of circulating progesterone. Enzymes primarily responsible for progesterone catabolism are from the cytochrome P450 2C and 3A sub-families [7,8]. Researchers have recently investigated the influence of insulin on progesterone catabolism in the context of eventually enabling methods for nutritionally manipulating progesterone clearance to reduce pregnancy loss from insufficient progesterone levels. Previous studies in vitro have indicated that insulin down-regulates the rate of progesterone catabolism in hepatocytes [34]. Lemley et al. [23] demonstrated increased circulating insulin in ewes within an hour following gavage of a highly gluconeogenic substrate, which was associated with lower cytochrome P450 2C and 3A activity in liver tissue collected by biopsy at one hour following gavage. This study reported changes in activity of progesterone catabolic enzymes induced by orally administered gluconeogenic substrate, providing a relatively physiologic, in vivo example of the potential effect of nutrition on circulating progesterone. In contrast, there was no difference in the activity of these progesterone catabolizing enzymes in the present study, even though these NR offspring have been previously shown to be relatively hyperinsulinemic and have enhanced insulin output in response to glucose challenge [35]. The tendency for reduced progesterone content of luteal tissue at the mid-luteal phase of the estrous cycle in NR offspring suggests a compromised ability for progesterone production in the ovaries of these NR offspring. Luteal tissue insufficiency may be a result of programming of ovarian function in early gestation. Previous results from this same ewe model of maternal nutrient restriction have shown greater oxidative damage to the DNA of fetal oogonia at mid-gestation [5]. Damaged nuclear material of fetal gametes could lead to lowered oocyte, follicular and, eventually, luteal quality, which could explain the differences in luteal progesterone content observed here during old age in NR offspring. While this deficit in progesterone secretion was associated with reduced conception rates during the first breeding year, no further decreases in fertility were observed during subsequent breeding seasons.

Vascular development and subsequent blood flow through the CL are vital to luteal function and progesterone secretion. Luteal blood flow in the bovine has been shown to be a more appropriate indicator of luteal progesterone secretion than size [36]. Angiogenic factors, which mediate capillary development and proliferation within the CL are thus speculated to play a major role in its development and progesterone secretory function. While the angiogenic factors VEGF, FGF, ANG-1 and ANG-2 are known to mediate vascular development within the CL [33,34], there were no group differences in expression of these factors in luteal tissue of ewes examined in the study. This may be due to the known variation in the expression of these angiogenic factors throughout luteal lifespan. mRNA expression of VEGF and its receptor VEGF receptor 2 has been shown to be elevated during the early luteal phase in ewes followed by a significant decline during the mid to late luteal phase [37]. Similarly, FGF has also been shown to exhibit elevated mRNA expression during the early luteal phase in CL tissue, decrease during the mid-luteal phase and then increase again in late luteal phase CL tissue [38]. As luteal blood flow was not measured in this experiment, it is possible that there are differences between groups even in the absence of differences in angiogenic factor expression.

Progesterone is produced by CL using cholesterol as substrate through the steroidogenesis pathway. The enzyme StAR is a rate-limiting enzyme in steroidogenesis because it is responsible for transporting cholesterol to the mitochondria from the cytoplasm. Another important enzyme is P450scc; it cleaves the cholesterol side chain to form pregnenolone in the mitochondrial matrix; then 3β-HSD converts pregnenolone into progesterone in the smooth endoplasmic reticulum. In the current study, both StAR and P450scc mRNA expression were reduced. Protein expression of P450scc was reduced and StAR protein level tended to be lower in offspring exposed to maternal nutrient restriction during early to mid-gestation. Bernal et al. [39] have recently shown that maternal undernutrition throughout pregnancy decreased ovarian follicle number in rat offspring, but steroidogenic enzyme 3β-HSD mRNA expression was not altered in these rat offspring. Consistent with their result, 3β-HSD mRNA and protein expression in NR offspring was not different from C offspring in the present study. Bernal et al. [39] have also demonstrated increased ovarian oxidative stress evidenced by significantly increased PRX-3 mRNA expression in rat adult offspring born to undernourished dams during pregnancy. Reactive oxygen species have been known to inhibit steroidogenesis by blocking cholesterol transport to mitochondria leading to reduced progesterone production in the ovary [15,16]. PRX-3 is a mitochondrial member of antioxidant thioredoxin peroxidases that reduce mitochondrial hydrogen peroxide (H_2_O_2_) [17]. We found increased PRX-3 mRNA expression in NR offspring compared with C offspring. The observed reduction in steroidogenic enzyme expression in CL may be due to, at least partly, increased ovarian oxidative stress. However, it is also possible that mitochondrial numbers in CL was reduced in NR offspring compared to C offspring since both increased oxidative stress in mitochondria and/or reduced number of mitochondria in CL will result in decreased steroidogenic enzyme expression observed in NR offspring. Although the small sample size was a disadvantage in this longitudinal study, the comprehensive analysis of progesterone catabolic pathways, angiogenic factor expressions in the ovary and P4 synthesis pathway provide evidence for a permanent programming effect of maternal undernutrition on offspring CL function until later age.

## Conclusions

The present study has provided evidence for a permanently programmed effect of maternal undernutrition during early to mid-gestation on offspring cyclic progesterone concentration resulting from an impaired luteal progesterone secretion. While such alterations in progesterone levels were associated with impaired fertility only in nulliparous animals, further investigation is needed to determine specific implications of lower progesterone concentrations in offspring of nutrient restricted pregnancies, as well as any potential link between progesterone and nutrient metabolism.

## Competing interest

The authors state that they have no competing interests.

## Authors’ contributions

NML, PWN and SPF designed the study. NML and LAG performed the research/data collection. NML, NT, YM, COL, and WJM conducted laboratory analyses. NML and NT performed data analyses, interpreted data and drafted manuscript. PWN and SPF provided financial support and significant editing of the manuscript. All authors read and approved the final manuscript.
